# TSC1 and TSC2 regulate cilia length and canonical Hedgehog signaling via different mechanisms

**DOI:** 10.1007/s00018-018-2761-8

**Published:** 2018-02-02

**Authors:** Thomas Rosengren, Lasse Jonsgaard Larsen, Lotte Bang Pedersen, Søren Tvorup Christensen, Lisbeth Birk Møller

**Affiliations:** 1 Applied Human Molecular Genetics, Clinical Genetic Clinic, Kennedy Center, Copenhagen University Hospital, Rigshospitalet, Gl. Landevej 7, 2600 Glostrup, Denmark; 2 Department of Biology, The August Krogh Building, University of Copenhagen, Universitetsparken 13, 2100 Copenhagen, Denmark

**Keywords:** TSC, mTOR, Autophagy, LC3b, Hedgehog signaling, TGF-β signaling, Primary cilia, WNT5a

## Abstract

Primary cilia are sensory organelles that coordinate multiple cellular signaling pathways, including Hedgehog (HH), Wingless/Int (WNT) and Transforming Growth Factor-β (TGF-β) signaling. Similarly, primary cilia have been implicated in regulation of mTOR signaling, in which Tuberous Sclerosis Complex proteins 1 and 2 (TSC1/2) negatively regulate protein synthesis by inactivating the mTOR complex 1 (mTORC1) at energy limiting states. Here we report that TSC1 and TSC2 regulate Smoothened (SMO)-dependent HH signaling in mouse embryonic fibroblasts (MEFs). Reduced SMO-dependent expression of *Gli1* was demonstrated in both *Tsc1*^−/−^ and *Tsc2*^−/−^ cells, and we found that *Tsc1* is required for TGF-β induced phosphorylation of SMAD2/3 and subsequent expression of the HH signaling effector and transcription factor GLI2. Hedgehog signaling was restored in *Tsc1*^−/−^ cells after exogenous expression of *Gli2*, whereas rapamycin restored HH signaling in *Tsc*2^−/−^ cells. Furthermore, we observed that *Tsc1*^−*/*−^ MEFs display significantly elongated cilia, whereas cilia in *Tsc2*^−*/*−^ MEFs were shorter than normal. The elongated cilium phenotype of *Tsc1*^−/−^ MEFs is likely due to increased mTORC1-dependent autophagic flux observed in these cells, as both the autophagic flux and the cilia length phenotype was restored by rapamycin. In addition, ciliary length control in *Tsc1*^−/−^ MEFs was also influenced by reduced expression of *Gli2,* which compromised expression of *Wnt5a* that normally promotes cilia disassembly. In summary, our results support distinct functions of *Tsc1* and *Tsc2* in cellular signaling as the two genes affect ciliary length control and HH signaling via different mechanisms.

**Electronic supplementary material:**

The online version of this article (10.1007/s00018-018-2761-8) contains supplementary material, which is available to authorized users.

## Introduction

Mutations in the tumor suppressor genes encoding TSC1 (Hamartin) and TSC2 (Tuberin) cause a multisystemic tumor syndrome termed tuberous sclerosis complex (TSC). TSC is an autosomal dominant genetic disorder of high penetrance with a prevalence of 1:10,000 [1] and is characterized by widespread dysplastic and neoplastic lesions [2]. TSC1 and TSC2 were originally found to form a heterodimeric complex that acts as a switch for turning off mTOR signaling by inactivating the mTOR complex 1 (mTORC1). Active mTORC1 phosphorylates the eukaryotic initiation factor 4E-binding protein-1 (4E-BP1) and 40S ribosomal protein S6 kinase 1 (S6K1) to promote protein synthesis [3, 4]. The phosphorylated form of 40S ribosome protein S6 is thus a marker for mTORC1 activity. The heterodimeric TSC complex negatively regulates mTORC1 activity via the GTPase activity of TSC2, towards the small G-protein RHEB (Ras homologue enriched in brain) [5]. The mTORC1 signaling cascade receives inputs from various upstream cues related to the metabolic and nutritional status of the cell and translates these into cell growth [4, 6]. In the presence of nutrients, mTORC1 is activated and promotes cell growth, including protein synthesis and energy storage. Conversely, during starvation mTORC1 is inhibited and autophagy is induced by AMP-activated protein kinase (AMPK), leading to generation of intracellular nutrients and energy during degradation of non-functional or non-essential organelles or protein aggregates [4, 6], in turn contributing to cell survival. During starvation AMPK promotes autophagy by activating ULK1 via phosphorylation at Ser317, Ser555, and Ser777, whereas in the presence of nutrients mTORC1 suppresses autophagy by hampering interaction between AMPK and ULK1 through phosphorylation of ULK1 at Ser757 [7]. In addition, the TSC complex activates mTOR complex 2 (mTORC2) [8], which regulates the cytoskeleton through its stimulation of F-actin stress fibers and Rho GTPases [9]. TSC1 and TSC2 also play a role in regulating pathways other than mTORC1/2. For example, TSC inactivation has been shown to cooperate with non-canonical Smoothened (SMO)-independent Hedgehog (HH) signaling to drive tumor growth in murine cerebellar granule neuron precursors [10]. Furthermore, TSC1 was demonstrated to be required for the proper activation of the transforming growth factor-β (TGF-β)-SMAD2/3 pathway in HeLa cells [11]. Despite these advances, the roles of TSC1 and TSC2 in regulating signaling pathways independently of mTORC1/2 remain poorly understood.

Primary cilia are microtubule-based sensory organelles that emanate from the centrosomal mother centriole/basal body at the surface of many quiescent vertebrate cell types [12]. These cilia play critical roles in the coordination of multiple cellular signaling pathways, which are regulated by diverse chemical, physical, and morphogenetic cues during development and in tissue homeostasis [13, 14]. Defects in ciliary assembly, maintenance, and function are, therefore, associated with severe developmental defects and diseases, known as ciliopathies, which include congenital heart defects, skeletal dysplasia, renal diseases, cerebral anomalies, obesity, tumorigenesis, and cancer [12, 15–19]. The cellular signaling systems that are operated by primary cilia include HH [20, 21], Wingless/Int (WNT) [22–24], platelet-derived growth factor receptor alpha (PDGFR-α) [25–27], TGF-β [28, 29], Notch [30], Hippo [31], and mTOR signaling [32, 33]. Recent studies have also indicated a functional connection between autophagy and ciliogenesis and/or cilia-coordinated signaling, but there is currently no consensus on whether increased autophagy leads to longer or shorter cilia [34, 35].

The vertebrate HH signaling pathway is one of the best studied ciliary signaling pathways and is essential for embryonic development and the maintenance of multiple organs [36, 37]. The 12-pass transmembrane receptor patched homolog 1 (PTCH1) is localized to the ciliary membrane in the absence of HH and prevents ciliary localization of SMO, a class F, G-protein coupled receptor that is required for the activation of GLI transcription factors. In this “OFF” stage, GLI2 and GLI3 are proteolytically cleaved to form repressor forms (GLI-R), which block transcriptional activation of target genes in HH signaling. The binding of HH to PTCH1 induces ciliary exit of PTCH1 causing concomitant ciliary enrichment of SMO, which results in decreased ciliary cAMP production, and in stabilization of full-length forms of GLI2 and GLI3 (GLI2-FL and GLI3-FL) in the cilium followed by translocation of these proteins to the nucleus in their activated forms (GLI2-A and GLI3-A) to induce transcriptional activation of target genes, including *Gli1* and *Ptch1* [38–42].

Compared to HH signaling, less is known about the role of primary cilia in mTOR signaling. TSC1 was shown to localize to the base of the cilium [43], and its loss in zebrafish was associated with left–right asymmetry defects in addition to ciliary elongation [33]. An aberrant cilia phenotype was also reported in murine cells where loss-of-function mutations in either *Tsc1* or *Tsc2* induced elongation of primary cilia [43, 44]. In addition, primary cilia were shown to regulate mTORC1 activity through the LKB1-AMPK pathway in murine kidney cells [32]. While the heterodimeric TSC1-TSC2 complex plays a crucial regulatory role in mTORC1 activity, the need to evaluate the separate functions of TSC1 and TSC2 is evident. Although evidence supports the interdependence of TSC1 and TSC2 in the regulation of mTORC1 [45], more than 50 proteins have been reported to interact with TSC1 and/or TSC2 [46], suggesting that both proteins function in other cellular contexts independent of each other.

Here we report that both *Tsc1*^−*/*−^ and *Tsc2*^−*/*−^ mouse embryonic fibroblasts (MEFs) affect ciliary length control and cellular signaling pathways, but via different mechanisms. First, we found that *Tsc1*^−*/*−^ cells exhibited elongated cilia, which we show is due to dysregulated autophagy and mTORC1 activity as well as reduced GLI2-mediated expression of *Wnt5a*. In contrast, we observed significantly shortened cilia in *Tsc2*^−*/*−^ cells. Second, while both *Tsc1*^−*/*−^ and *Tsc2*^−*/*−^ MEFs display impaired SMO-dependent HH signaling, TSC2 affects HH signaling via mTORC1, whereas TSC1 regulates HH signaling via the TGF-β-SMAD2/3 pathway, which in turn regulates expression of the GLI2 transcription factor required for HH target gene expression. Collectively, our results indicate that TSC1 and TSC2 affect ciliary length control and canonical HH signaling via different mechanisms.

## Results

### Primary cilia are elongated and shortened in *Tsc1*^−*/*−^ and *Tsc2*^−*/*−^ MEFs, respectively

**Fig. 1:**
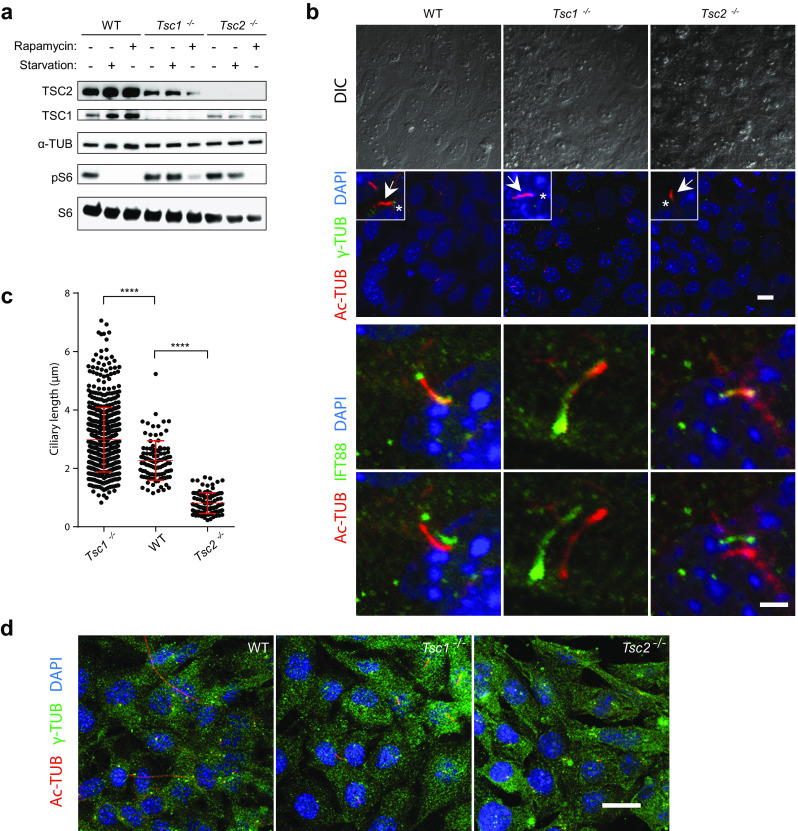
Primary cilia are elongated in *Tsc1*^−*/*−^ but shortened in *Tsc2*^−*/*−^ cells. **a** SDS-PAGE and WB analyses of cell lysates from WT, *Tsc1*^−*/*−^ and *Tsc2*^−*/*−^ MEFs cultured in normal (-starvation) or starvation medium (0.5% FBS) for 48 h, using antibodies against TSC1, TSC2, S6 and phospho-S6 (pS6) as indicated, with antibody against α-tubulin (α-TUB) as loading control. Note the presence of pS6 in serum-starved *Tsc1*^−*/*−^ and *Tsc2*^−*/*−^ cells. **b** IFM analysis of primary cilia in WT*, Tsc1*^−*/*−^ and *Tsc2*^−*/*−^ MEFs cultured in starvation medium (0.5% FBS) for 48 h, to induce ciliogenesis. Cilia (arrows) were labeled with anti-acetylated α-tubulin (Ac-TUB) and anti-IFT88 antibodies and the ciliary base/centrosomes (asterisks) were labeled with anti-γ-tubulin (γ-TUB) antibody. Nuclei were visualized with differential interference contrast (DIC) microscopy or DAPI staining. Lower panels show merged and shifted overlays of Ac-TUB- and IFT88-labeled cilia. Scale bar: 2 µM. **c** Quantification of cilia lengths for experiment shown in **b**. Three hundred cilia (Ac-TUB) from three independent experiments were used for quantification. **d** IFM analysis of WT*, Tsc1*^−*/*−^ and *Tsc2*^−*/*−^ MEFs labeled with DAPI, Ac-TUB and IFT88 for cell size comparison. Nuclei were visualized with DAPI staining. Scale bar: 5 µM

At low nutrient levels, the TSC1-TSC2 complex downregulates mTORC1 activity so that the loss of either protein is associated with constitutive activation of mTOR signaling [47, 48]. To confirm this, *Tsc1*^−*/*−^ and *Tsc2*^−*/*−^ MEFs were starved (0.5% fetal bovine serum, FBS) for 48 h and analyzed by SDS-PAGE and western blotting (WB) for the expression of *Tsc1* and *Tsc2,* and the phosphorylation status of S6 (pS6), which is a marker for mTORC1 activity (Fig. 1a). As expected, TSC1 and TSC2 were absent in *Tsc1*^−*/*−^ and *Tsc2*^−*/*−^ MEFs, respectively. It has previously been demonstrated that TSC1 stabilizes TSC2 by protecting TSC2 from ubiquitination and proteosomal degradation [49]. This concurs with the lower level of TSC2 observed in *Tsc1*^−*/*−^ MEFs (Fig. 1a). Furthermore, phosphorylation of S6 (pS6) was detectable in both *Tsc1*^−*/*−^ and *Tsc2*^−*/*−^ MEFs upon serum depletion, demonstrating constitutive mTORC1 activity. This activity was hampered by treatment with the mTORC1 inhibitor, rapamycin, verifying that phosphorylation of S6 is mTORC1-dependent (Fig. 1a).

**Fig. 2:**
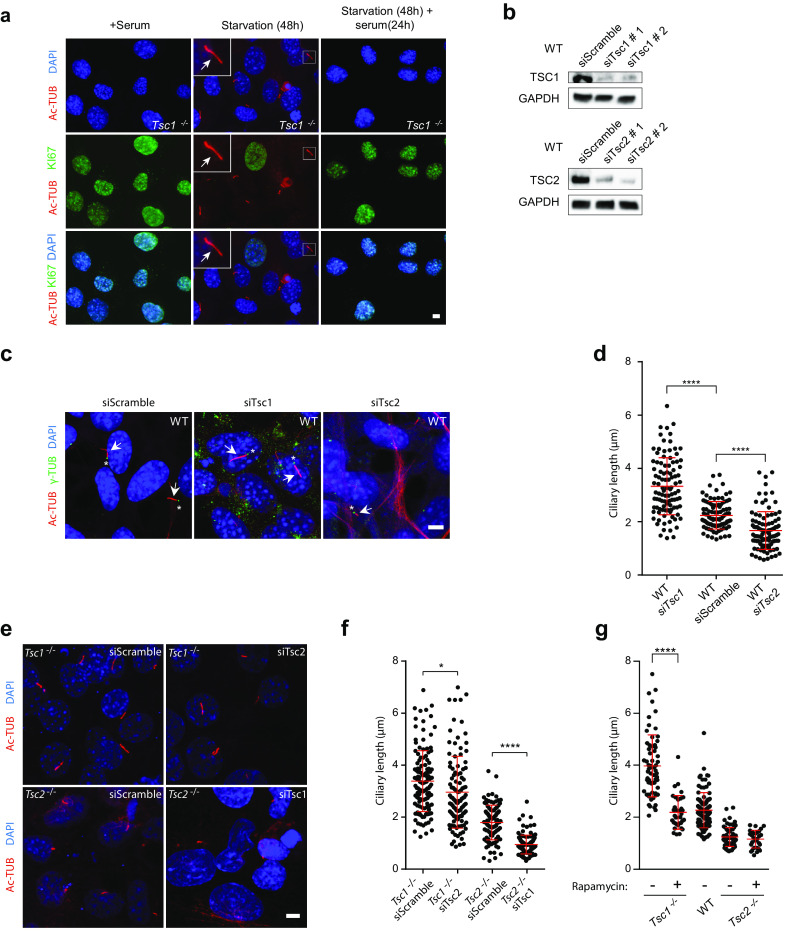
Rapamycin treatment rescues the ciliary length phenotype of *Tsc1*^−*/*−^ cells. **a** IFM analysis of the expression of the proliferation marker KI67 and formation of primary cilia (arrows), labeled with anti-acetylated α-tubulin (Ac-TUB) antibody, in *Tsc1*^−*/*−^ MEFs cultured in normal serum medium (left panels), in starvation medium (0.5% FBS) for 48 h (middle panels, with few proliferating cells), or after 48 h starvation followed by serum re-stimulation for 24 h (right panels, with many proliferating cells). **b** WB analysis of WT MEFs subjected to siRNA-mediated knock down of *Tsc1* or *Tsc2*. WT MEFs were analyzed 48 h after transfection with siTsc1, siTsc2 or siScramble (negative control). GAPDH was used as loading control. **c** IFM analysis of primary cilia in WT MEFs after knock down of *Tsc1* or Tsc2. Cilia (arrows) were labeled with Ac-TUB antibodies and the ciliary base/centrosomes (asterisks) were labeled with anti-γ-tubulin antibody (γ-TUB). Nuclei were visualized with DAPI staining. Scale bar: 5 µM. **d** Quantification of ciliary lengths for experiment shown in **c**. Three hundred cilia from three independent experiments were used for quantification; error bars represent SEM. **e** IFM analysis of primary cilia (Ac-TUB) in *Tsc1*^−*/*−^ and *Tsc2*^−*/*−^ MEFs subjected to siRNA-mediated knock down followed by culturing in starvation medium (0.5% serum) for 48 h (24 h after transfection) to induce cilia formation. Nuclei were visualized with DAPI staining. Scale bar: 5 µM. **f** Quantification of ciliary lengths for experiment shown in **e**. One hundred cilia from three independent experiments were used for quantification; error bars represent SEM. **g** Quantification of ciliary lengths labeled with Ac-TUB antibody in WT, *Tsc1*^−*/*−^ and *Tsc2*^−*/*−^ MEFs cultured in low 48 h in the presence or absence of rapamycin

Next, we evaluated the consequence of TSC1 and TSC2 depletion on the frequency and length of primary cilia during serum starvation. To this end, *Tsc1*^−*/*−^ and *Tsc2*^−*/*−^ MEFs were serum starved (0.5% FBS) for 48 h to induce growth arrest and formation of primary cilia, and subsequently analyzed by immunofluorescence microscopy (IFM) with antibodies against γ-tubulin that marks the ciliary base, acetylated α-tubulin that marks the cilium, and IFT88, which is component of the intraflagellar transport system and marks both the ciliary base and the cilium (Fig. 1b). While the frequency of ciliated cells appeared to be unaffected by depletion of either TSC1 or TSC2 (wild type (WT): 53.6 ± 10.4; *Tsc1*^−*/*−^: 56.6 ± 2.3; *Tsc2*^−*/*−^: 60.7 ± 5.9), we found that cilia in *Tsc1*^−*/*−^ MEFs were significantly longer with an average length of 2.98 μm as compared to WT MEFs that displayed an average ciliary length of 2.20 μm (Fig. 1b, c), which is in accordance with previous observations [43]. In contrast, we observed that the length of primary cilia in *Tsc2*^−*/*−^ cells was significantly decreased with a mean length of 0.74 μm (Fig. 1c). The observed ciliary length phenotypes of the mutant cells were not an indirect effect of increased cell size [50], since microscopic analysis showed that the sizes of mutant and wild type cells were largely similar (Fig. 1d). Furthermore, the ciliary length phenotype of the *Tsc1*^−*/*−^ MEFs did not seem to be secondary to cell cycle defects, as judged by staining with antibody against acetylated tubulin and KI67, a potent proliferation marker; primary cilia were only induced in quiescent cells and re-addition of serum lead to cell cycle re-entrance as expected (Fig. 2a).

We then asked if exogenous expression of *TSC1* and *TSC2* could rescue the ciliary length phenotype of *Tsc1*^−*/*−^ and *Tsc2*^−*/*−^ cells. While transfection with TSC1-encoding plasmid (pTSC1) partly restored the ciliary length in *Tsc1*^−*/*−^ cells, only minor effect was observed by transfection of *Tsc2*^−*/*−^ cells with TSC2-encoding plasmid (pTSC2) (Supplementary Figure 1a, b). The latter result could be due to a toxic effect of the transfection and/or aberrant production of a smaller protein product (Supplementary Figure 1c, d). The transfection efficiency, measured by transfection with pGFP plasmid, is 14–15% (Supplementary Figure 1e). As an alternative approach to confirm the ciliary length phenotypes of the *Tsc1*^−*/*−^ and *Tsc2*^−*/*−^ cells, we used siRNA to deplete TSC1 or TSC2 from WT MEFs (Fig. 2b). Using this approach, we found that the siRNA-depleted WT cells displayed similar ciliary lengths as observed in *Tsc1*^−*/*−^ and *Tsc2*^−*/*−^ cells, respectively (Fig. 2c, d), confirming the mutant cell phenotypes. To test the dominancy of the two genes, we furthermore subjected *Tsc1*^−*/*−^ cells to siRNA-mediated depletion of TSC2, and vice versa (Fig. 2e; Supplementary Figure 2). In both cases, a reduction in the ciliary length was observed, indicating that the absence of *Tsc2* is dominant for the resulting phenotype (Fig. 2f).

Finally, we induced cilia formation in starvation medium (0.5% FBS) in combination with rapamycin and found that the ciliary length phenotype of *Tsc1*^−*/*−^ MEFs was rescued by this treatment, while rapamycin had no observable effect on the ciliary length in *Tsc2*^−*/*−^ MEFs (Fig. 2g). These results support the conclusion that the increased ciliary length in *Tsc1*^−*/*−^ MEFs is associated with aberrant activity of mTORC1. Consistent with Hartman et al. [43], the ciliation frequency in WT, *Tsc1*^−*/*−^ and, *Tsc2*^−*/*−^ cells did not appear to be affected by rapamycin.

### *Tsc1*^−*/*−^ MEFs exhibit increased mTORC1-dependent autophagy

**Fig. 3:**
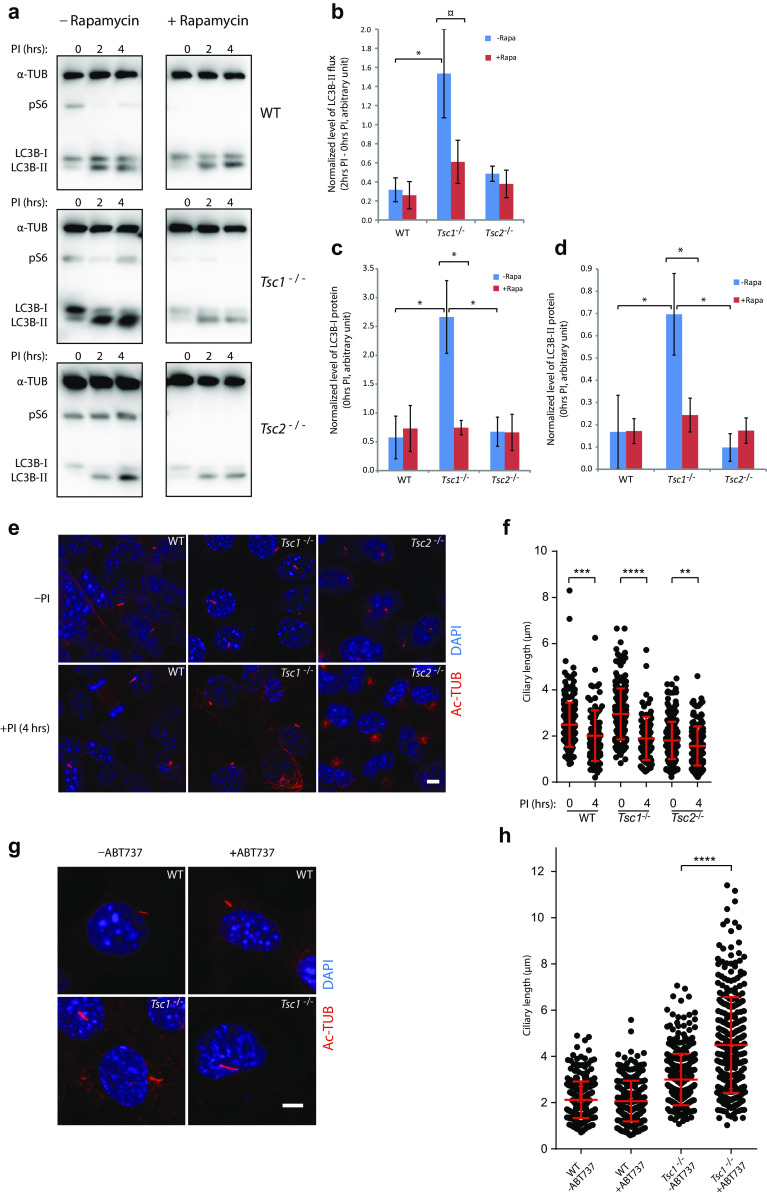
Loss of *Tsc1* leads to increased mTORC1-dependent autophagic activity. **a** SDS-PAGE and WB analyses on the level of LC3B and phosphoS6 (pS6) in WT, *Tsc1*^−*/*−^ and *Tsc2*^−*/*−^ MEFs starved (0.5% FBS) for 48 h in the absence or presence of rapamycin (Rapa) (24 h) followed by treatment with lysosomal protease inhibitors (PI) as indicated. α-tubulin (α-TUB) was used as loading control. **b** Normalized LC3B-II flux for experiments shown in **a** as measured by the LC3B-II protein levels after 2 h PI treatment subtracted the protein levels at 0 h. **c** Normalized LC3B-I protein levels for experiments shown in **a** before PI treatment. **d** Normalized LC3B-II protein levels for experiments shown in **a** before PI treatment. **b**–**d** Quantifications of LC3B-I/II protein levels were performed by densitometric analysis and normalized to α-TUB, and to a control sample loaded on all gels to correct for blotting efficiency. Error bars represent SEM (*n* = 3). Recalculating of the LC3B-II flux relative to the amount of LC3B-I eliminated the difference between the cell lines and the effect of rapamycin on the *Tsc1*^−*/*−^ cells (WT: 0.506 ± 0.087/0.584 ± 0.864; *Tsc1*^−*/*−^: 0.6261 ± 0.314/0.864 ± 0.443; *Tsc2*^−*/*−^: 0.673 ± 0.253/0.662 ± 0.314. No statistic significant differences were obtained). **e** IFM analysis of primary cilia labeled with anti-γ-tubulin (γ-TUB) antibody in WT, *Tsc1*^−*/*−^ and *Tsc2*^−*/*−^ MEFs after 4 h PI treatment. Scale bar: 5 µM. **f** Quantification of ciliary lengths for experiment shown in **e**. One hundred cilia from three independent experiments were used for quantification; error bars represent SEM. **g** IFM analysis of primary cilia labeled with γ-TUB antibody in serum-deprived (48 h) WT and *Tsc1*^−*/*−^ MEFs in the presence and in the absence of the autophagy inducer ABT-737. Scale bar: 5 µM. **h** Quantification of ciliary lengths for experiment shown in **g**. One hundred cilia from two independent experiments were used for quantification; error bars represent SEM

Several studies have shown that autophagy regulates ciliary length [34, 35, 51–55]. To test for differences in autophagic activity between *Tsc1*^−*/*−^ and *Tsc2*^−*/*−^ MEFs, we performed an autophagic flux assay, measuring the net flux of LC3B-II in starvation medium (0.5% FBS), stimulating both autophagy and formation of primary cilia (Fig. 3a). LC3B is expressed as a pro-LC3B protein, which is subsequently cleaved by removing the C terminus to produce the cytosolic form, LC3B-I. The cytosolic form is conjugated with phosphatidylethanolamine to form the membrane-bound LC3B-II, which is attached to the autophagosome membrane reflecting maturation of the autophagosomes. After fusion of the autophagosomes with lysosomes, LC3B-II is degraded or released as LC3B-I through delipidation. Turnover of LC3-II thus reflects the autophagic activity [35]. The net flux and the amount of LC3B-I and LC3B-II were significantly increased in the *Tsc1*^−*/*−^ cells compared to the WT and *Tsc2*^−*/*−^ cells (Fig. 3b–d). As the cilia length in *Tsc1*^−/−^ cells was reduced in the presence of rapamycin (Fig. 2g), we investigated the effect of rapamycin on autophagy. Rapamycin reduced the net flux, and the amount of LC3B-I and LC3B-II in the *Tsc1*^−*/*−^ cells to levels comparable to that of WT cells. No effect of rapamycin on LC3B was observed for the WT or the *Tsc2*^−*/*−^ cells (Fig. 3a–d). This indicates that the long cilia phenotype in *Tsc1*^−*/*−^ cells may be a result of increased net flux. Compared to *Tsc2*^−*/*−^ and WT cells, *Tsc1*^−*/*−^ cells seem to contain larger amounts of LC3B protein. Recalculating the efflux relative to the amount of LC3B-I eliminated the difference between the cell lines and the effect of rapamycin on the *Tsc1*^−*/*−^ cells (legend Fig. 3d). Thus, the increased amount of LC3B-II in *Tsc1*^−*/*−^ cells correlates with the increased LC3B-I level.

To test the effect of the autophagic activity on the ciliary length more directly, we investigated the ciliary length in the *Tsc1*^−/−^ cells after treatment with lysosomal protease inhibitors for 4 h to inhibit autophagy, and found a profound reduction (Fig. 3e, f). However, a significant reduction in the ciliary length in both the *Tsc2*^−/−^ and the WT cells was also observed (Fig. 3f). In contrast, treatment with the autophagy inducer ABT-737 [56] strongly induced ciliary elongation in the *Tsc1*^−/−^ cells, whereas no effect was observed in the WT cells (Fig. 3g, h). In summary, these results indicate that autophagy initiated by serum starvation is important for the growth of the primary cilium in general, whereas increased autophagy specifically causes ciliary lengthening in the *Tsc1*^−/−^ cells.

### *Tsc1*^−*/*−^ and *Tsc2*^−*/*−^ MEFs display impaired SMO-dependent HH signaling

**Fig. 4:**
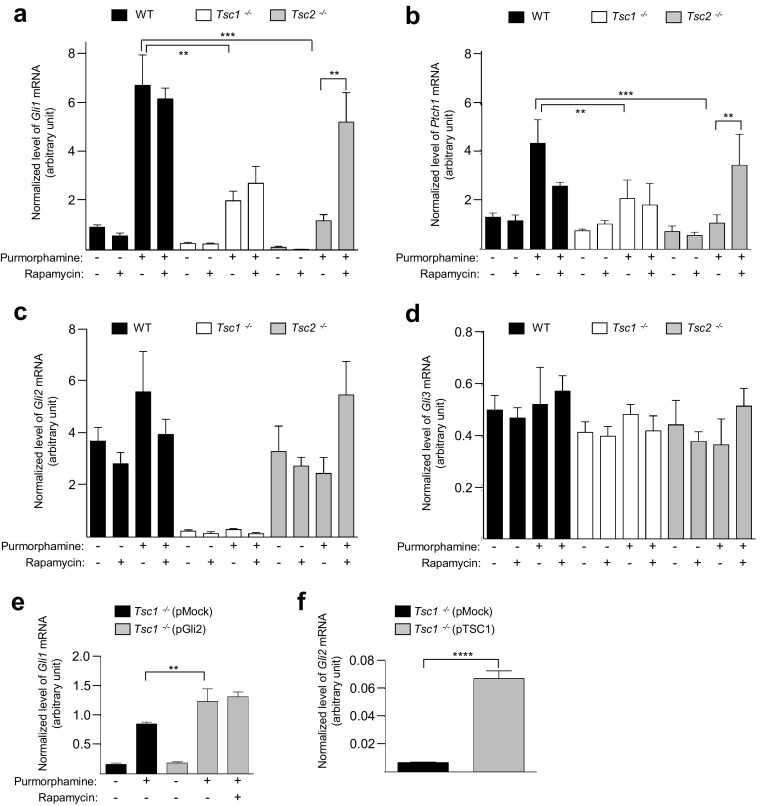
Loss of *Tsc1* or *Tsc2* is associated with impaired HH signaling. **a**–**d** WT, *Tsc1*^−*/*−^ and *Tsc2*^−*/*−^ MEFs were starved (0.5% FBS) for 48 h to induce cilia formation in the presence or absence of rapamycin. Purmorphamine (5 µM) was administered to the medium for the final 24 h to stimulate HH signaling before mRNA isolation and qPCR analysis. Expression profiles of the target genes were normalized to the amount of endogenous *Tbp* mRNA. Normalized expression profiles of **a** *Gli1*, **b** *Ptch1.* Error bars represent SEM (*n* = 5). Normalized expression profiles of **c**  *Gli2* and **d**  *Gli3*. **e** Normalized expression profiles of *Gli1* in *Tsc1*^−*/*−^ MEFs transfected with 2 µg plasmid (pMock (empty vector) or pGli2) and cultured in starvation medium (0.5% FBS) for 48 h (24 h after transfection) with purmorphamine administration (for the last 24 h), as indicated. Error bars represent SEM (*n* = 3). The reduced signal in **e** compared to **a** might be due to transfection (pMock included) of the cells. **f** Normalized expression profiles of *Gli2* in *Tsc1*^−*/*−^ MEFs transfected with 1 µg plasmid (pMock or pTSC1) and starved (0.5% serum) for 48 h (24 h after transfection). Error bars represent SEM (*n* = 3)

Some evidence points to a possible link between the HH and mTOR signaling, since a combination therapy inhibiting both pathways results in an enhanced therapeutic effect compared to inhibiting either pathway alone [57]. Therefore, we speculated whether HH signaling is altered in *Tsc1*^−*/*−^ and *Tsc2*^−*/*−^ MEFs. To monitor HH signaling, we initially performed quantitative RT-PCR (qPCR) analysis of the expression of the HH target genes, *Gli1* and *Ptch1* in cells starved (0.5% FBS) for 48 h to induce cilia formation. To activate canonical HH signaling, the SMO agonist purmorphamine [58] was added for the last 24 h of cultivation. Furthermore, cells were cultured in the presence and in the absence of rapamycin to evaluate the role of mTORC1 in purmorphamine-induced expression of the target genes. As shown (Fig. 4a, b), purmorphamine-induced expressions of *Gli1* and *Ptch1* were significantly reduced in both *Tsc1*^−*/*−^ and *Tsc2*^−*/*−^ MEFs as compared to WT cells. However, rapamycin treatment restored the mRNA levels to WT levels only in *Tsc2*^−*/*−^ MEFs, indicating that defects in HH signaling are associated with increased mTORC1 activity in *Tsc2*^−*/*−^ MEFs. Since rapamycin treatment abrogated the elongated cilia phenotype observed in *Tsc1*^−*/*−^ MEFs (Fig. 2g), we further conclude that the long cilia phenotype observed in these cells (Fig. 1) is not directly caused by aberrant HH signaling or vice versa.

To further investigate the mechanisms by which HH signaling is impaired in *Tsc1*^−*/*−^ and *Tsc2*^−*/*−^ MEFs, we then performed qPCR analysis of the mRNA levels of *Gli2* and *Gli3*, which are generally constitutively expressed in normal cells [59, 60]. In these experiments, we observed that the expression of *Gli2,* but not *Gli3*, is greatly reduced in *Tsc1*^−*/*−^ cells, both in the presence and absence of purmorphamine and rapamycin (Fig. 4c, d). Cellular fractionation and WB analysis confirmed the absence of detectable GLI2 in the nucleus of *Tsc1*^−*/*−^ cells (Supplementary Figure 3). This indicates that reduced expression of *Gli1* and *Ptch1* may be caused by the lack of a sufficient level of activator forms of GLI2 independent of mTORC1 activity. In contrast, *Tsc2*^−*/*−^ MEFs displayed normal expression levels of both *Gli2* mRNA and *Gli3* mRNA compared to WT cells (Fig. 4c, d). To determine whether decreased HH signaling in *Tsc1*^−*/*−^ MEFs can be explained by the loss of GLI2, we examined HH signaling in *Tsc1*^−*/*−^ MEFs in which a plasmid expressing GLI2 was introduced. In agreement with our hypothesis, exogenous expression of *Gli2* caused significantly elevated expression of *Gli1* in response to purmorphamine treatment in these cells (Fig. 4e; Supplementary Figure 4). We also evaluated the level of *Gli2* mRNA in *Tsc1*^−*/*−^ MEFs subjected to exogenous expression of *TSC1*, by transfecting the cells with the pTSC1 plasmid. In this case, we observed a ~ tenfold increase in the level of *Gli2* transcript, establishing a direct link between TSC1 and *Gli2* expression (Fig. 4f; Supplementary Figure 5). Finally, inhibition of mTORC1 activity by rapamycin did not affect the purmorphamine-induced increase in *Gli1* expression in *Tsc1*^−*/*−^ MEFs even after introducing exogenous *Gli2* (Fig. 4e). This indicates that TSC1 regulates HH signaling through the expression of *Gli2* and this regulation is independent of mTORC1 activity.

### TSC1 regulates HH signaling via a TGF-β-SMAD2/3-dependent pathway

**Fig. 5:**
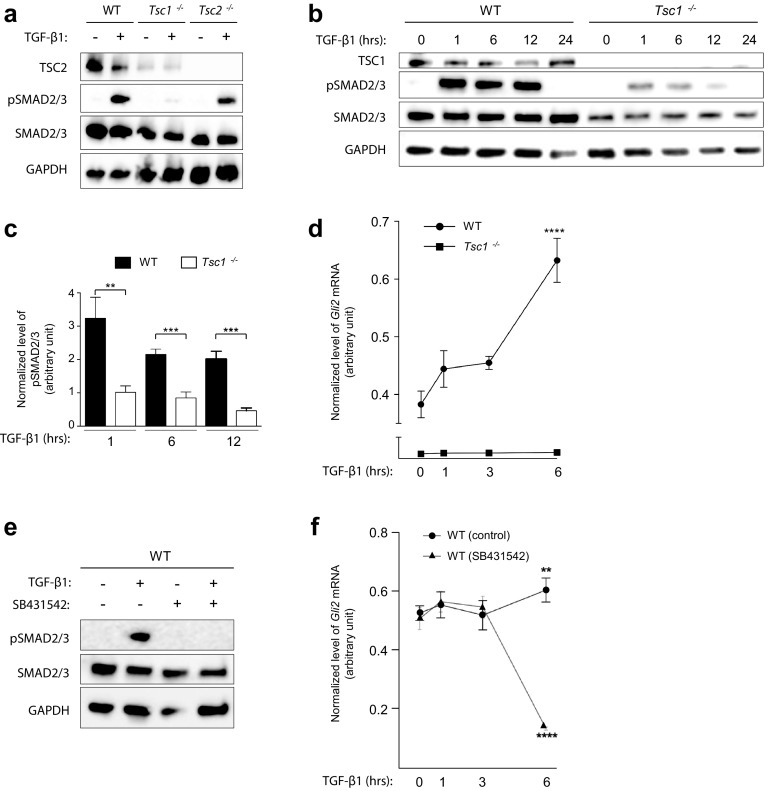
*Tsc1*^−*/*−^ cells display impaired TGF-β-SMAD2/3 signaling. **a** SDS-PAGE and WB analyses of WT, *Tcs1*^−*/*−^ and *Tsc2*^−*/*−^ MEFs serum starved (0.5% serum) for 48 h to induce cilia formation in the presence or absence of TGF-β1 for 45 min. Antibodies against TSC2, phospho-SMAD2/3 (pSMAD2/3) and SMAD2/3 were used as indicated. GAPDH was used as loading control. **b** SDS-PAGE and WB analyses of WT and *Tsc1*^−*/*−^ MEFs serum starved for 48 h in the presence of TGF-β1 for the indicated time points. **c** Quantification of relative levels of pSMAD2/3 normalized to total SMAD2/3 for experiment shown in **b**. Error bars represent SEM (*n* = 3). **d** Normalized expression profiles of *Gli2* mRNA in WT and *Tsc1*^−*/*−^ MEFs cultured to confluence in complete medium and subjected to TGF-β1 stimulation for the indicated time points before RNA isolation and qPCR. Error bars represent SEM (*n* = 3). **e** SDS-PAGE and WB analyses of WT MEFs cultured in serum deprived (0.5% FBS, 48 h) in the presence or absence of TGF-β1 and SB431542 for 45 min. **f** Normalized expression profiles of *Gli2* mRNA in WT MEFs cultured in complete medium and subjected to TGF-β1 in the presence or absence of SB431542 for the indicated time points. Error bars represent SEM (*n* = 3). Expression profiles of *Gli2* were normalized to endogenous *Tbp* mRNA

Previous studies have shown that primary cilia in fibroblasts coordinate canonical TGF-β signaling through clathrin-dependent endocytosis of activated receptors at the ciliary pocket followed by phosphorylation and activation of SMAD2/3 transcription factors in early endosomes around the ciliary base region [28]. Activated SMAD2/3 then forms a heterotrimeric complex with SMAD4, which translocates to the nucleus for expression of target genes. Interestingly, several reports have indicated that *GLI2* is a target gene for SMAD2/3 signaling in various human cell lines including fibroblasts [61, 62], and it has recently been shown that TSC1, independently of TSC2, is required for the association between TGF-β receptors and SMAD2/3, so that the knockdown of TSC1 causes an impaired phosphorylation and nuclear translocation of SMAD2/3 in HeLa cells [11]. We therefore speculated whether canonical TGF-β signaling is impaired in *Tsc1*^−*/*−^ MEFs, leading to a reduced expression of *Gli2*. To address this, we investigated SMAD2/3 phosphorylation in response to TGF-β1 stimulation. In contrast to WT and *Tsc2*^−*/*−^ MEFs, SMAD2/3 phosphorylation was significantly reduced in *Tsc1*^−*/*−^ MEFs after 1 h of stimulation with TGF-β1 (Fig. 5a), showing that TSC1, but not TSC2, is required for activation of canonical TGF-β signaling, as previously observed in HeLa cells [11].

To further examine whether the loss of TSC1 merely results in a delayed response to TGF-β1 stimulation, we evaluated the level of SMAD2/3 phosphorylation in *Tsc1*^−*/*−^ MEFs after 1, 6, 12, and 24 h of ligand treatment. These experiments showed that SMAD2/3 phosphorylation is reduced at all investigated time points (Fig. 5b, c), thereby substantiates the conclusion that TSC1 plays a critical role in the activation of SMAD2/3. No SMAD2/3 phosphorylation could be observed after 24 h (Fig. 5b) in agreement with a previous study showing that SMAD2/3 phosphorylation peaks after 1 h of ligand exposure, and declines over the next 8 h, to finally cease [63].

To investigate a potential relationship between impaired *Gli2* expression and SMAD2/3 phosphorylation in *Tsc1*^−*/*−^ MEFs, we carried out qPCR analysis on *Gli2* expression in response to TGF-β1 stimulation in these cells. Interestingly, an increase in *Gli2* expression was evident in WT MEFs for at least 6 h while no response in *Tsc1*^−*/*−^ MEFs was observed during this time interval (Fig. 5d). As a control for ligand specificity in receptor-mediated SMAD2/3 phosphorylation and requirement for TGF-β signaling in *Gli2* expression, SDS-PAGE, WB and qPCR analyses showed that administration of the TGF-β receptor antagonist, SB431542 [64], abolished SMAD2/3 phosphorylation (Fig. 5e) and greatly reduced the expression of *Gli2* in WT MEFs (Fig. 5f).

### Reduced *Gli2* expression affects *Wnt5a* expression resulting in elongated cilia in *Tsc1*^−*/*−^ MEFs

**Fig. 6:**
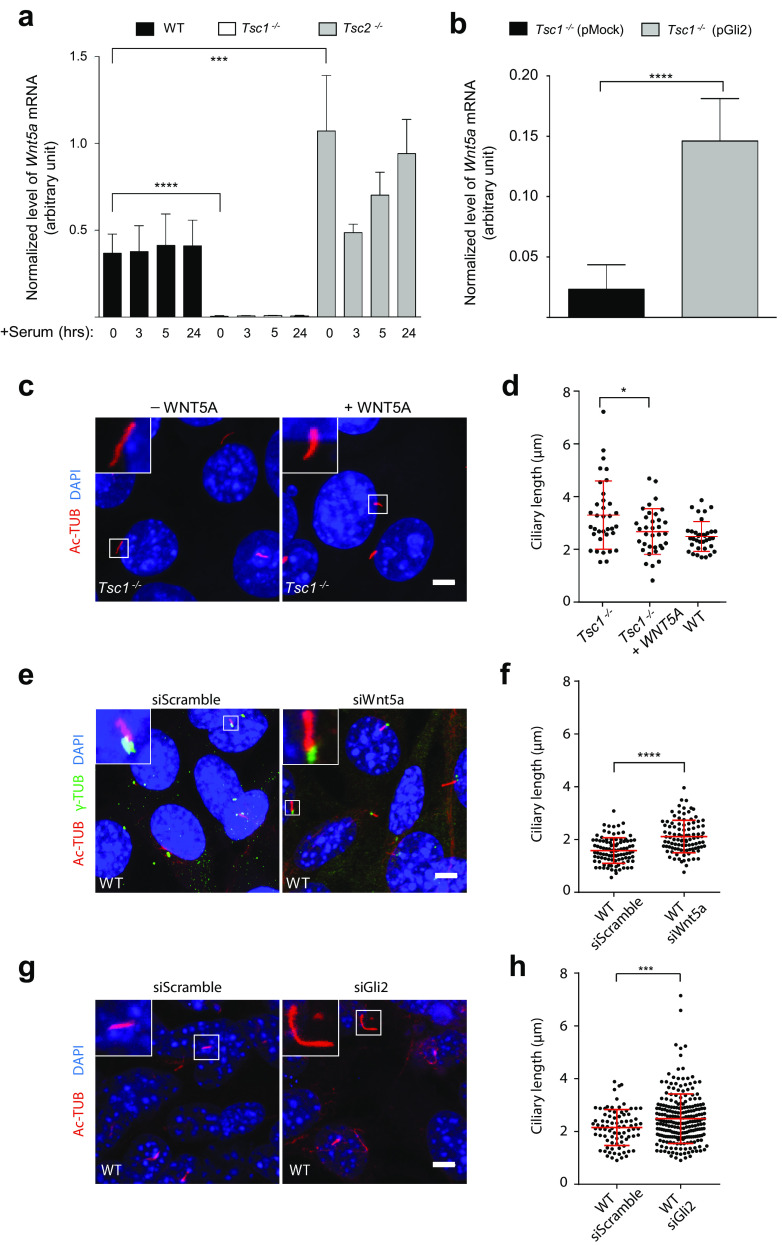
Cilia elongation in *Tsc1*^−*/*−^ cells is partly abrogated by WNT5a administration. **a** Normalized expression profiles of *Wnt5a* mRNA in WT*, Tsc1*^−*/*−^ and *Tsc2*^−*/*−^ MEFs starved (0.5% FBS) for 48 h followed by serum re-stimulation (10% FBS) for the indicated time points. Error bars represent SEM (*n* = 3). **b** Normalized expression profiles of *Wnt5a* mRNA in *Tsc1*^−*/*−^ MEFs transfected with 1 µg plasmid (pMock (empty vector) or pGli2) and cultured in complete medium for 48 h before RNA isolation and qPCR. Error bars represent SEM (*n* = 3). **a**, **b** Expression profiles of *Wnt5a* were normalized to endogenous *Tbp* mRNA. **c** IFM analysis of primary cilia in *Tsc1*^−*/*−^ MEFs serum deprived for 48 h in the presence and in the absence of recombinant WNT5A. Cilia were labeled with anti-acetylated α-tubulin (Ac-TUB) antibodies. Nuclei were visualized with DAPI staining. **d** Quantification of ciliary lengths for experiment shown in **c**. One hundred cilia from three independent experiments were used for quantification; error bars represent SEM (*n* = 3). **e** IFM analysis of primary cilia in WT MEFs transfected with either siScramble or siWnt5a for 24 h followed by serum deprivation for 48 h before analysis. Cilia were labeled with Ac-TUB antibody and the ciliary base/centrosome was labeled with anti-γ-tubulin (γ-TUB) antibody. Nuclei were visualized with DAPI staining. **f** Quantification of ciliary lengths for experiment shown in **e**. Three hundred cilia from three independent experiments were used for quantification; error bars represent SEM. **g** IFM analysis of primary cilia (Ac-TUB) in WT MEFs transfected with either siScramble or siGli2 for 24 h followed by serum deprivation for 48 h before analysis. Nuclei were visualized with DAPI staining. **h** Quantification of ciliary lengths for experiment shown in **g**. Two hundred cilia from three independent experiments were used for quantification; error bars represent SEM. Scale bars (**c**, **e**, **g**): 5 µM

The prominent differences in the lengths of primary cilia in *Tsc1*^−*/*−^ and *Tsc2*^−*/*−^ MEFs (Fig. 1b, c) suggest that signaling defects in these cells may also have an impact on ciliary length control. Both TGF-β and HH signaling have been shown to regulate the expression of the gene encoding the non-canonical WNT ligand, WNT5A [65–67], which is known to control ciliary length by inducing disassembly of the primary cilium [65, 66, 68, 69]. We therefore hypothesized that *Wnt5a* expression in *Tsc1*^−*/*−^ MEFs is compromised as a result of low *Gli2* expression. To investigate this, growth-arrested ciliated cells were subjected to re-addition of serum for different times to monitor the expression of *Wnt5a* by qPCR analysis during ciliary disassembly and cell cycle re-entry. As shown in Fig. 6a, the level of *Wnt5a* mRNA was clearly detected in WT and *Tsc2*^−*/*−^ MEFs, but almost undetectable in *Tsc1*^−*/*−^ MEFs (Fig. 6a), in agreement with our hypothesis. As *Wnt5a* has been shown also to be regulated by GLI3 [65], we furthermore investigated the GLI3-FL/GLI3-R ratio in WT and *Tsc1*^−*/*−^ MEFs before and after purmorphamine stimulation. Whereas a significant increase in the GLI3-FL/GLI3-R ratio could be observed in the WT MEFs as an effect of the purmorphamine stimulation, indicating higher amount of active GLI3, no effect could be observed in the and *Tsc1*^−*/*−^ cells (Supplementary Figure 3). Therefore, we cannot exclude that reduced formation GLI3-FL in conjunction with low levels of GLI2 is involved in the reduced amount of *Wnt5a* mRNA observed in the *Tsc1*^−*/*−^ MEFs.

To further investigate the relationship between the expression of *Gli2* and *Wnt5a*, we transfected *Tsc1*^−*/*−^ MEFs with the GLI2-encoding plasmid and found an increased expression of *Wnt5a* (Fig. 6b, Supplementary Figure 4). Indeed, stimulation with recombinant WNT5a restored the length of cilia in *Tsc1*^−*/*−^ MEFs to a level comparable to that of WT cells (Fig. 6c, d), supporting the conclusion that reduced expression of *Wnt5a* contribute to the elongated cilia phenotype of *Tsc1*^−*/*−^ MEFs. To confirm this observation, we used siRNA to knock down *Wnt5a* in WT MEFs and subsequently measured ciliary length after culturing the cells for 24 h in starvation medium (0.5% FBS). An increased ciliary length was observed, compared to cells transfected with an siRNA construct containing a scramble sequence (Fig. 6e, f; Supplementary Figure 6), confirming that the expression of *Wnt5a* promotes ciliary shortening. Moreover, siRNA-mediated knock down of *Gli2* in WT MEFs (Fig. 6g; Supplementary Figure 7) lead to ciliary lengthening (Fig. 6h), supporting that *Wnt5a* expression depends on GLI2.

It is possible that other factors than GLI2, including GLI3, may be involved in regulation of *Wnt5a* expression as elevated *Wnt5a* expression was observed in *Tsc2*^−*/*−^ MEFs (Fig. 6a), which is consistent with their short cilia phenotype, although the level of *Gli2* mRNA in *Tsc2*^−*/*−^ MEFs was comparable to the level in WT cells (Fig. 4c).

## Discussion

The heterodimeric TSC complex that consists of TSC1 (Hamartin) and TSC2 (Tuberin) plays a critical role in coordinating extracellular signals with intracellular energy status to control cell proliferation and growth [48]. However, the independent functions of the two TSC proteins are less well known. In this study, we investigated the cilia phenotypes in MEFs lacking either TSC1 or TSC2. While our observation of elongated cilia in *Tsc1*^−*/*−^ MEFs is in accordance with previous results [33, 43, 44], our observations of reduced ciliary length in *Tsc2*^−/−^ are not [43]. In a previous study performed by Hartman et al. [43], it was found that the cilia in both *Tsc1*^−*/*−^ and *Tsc2*^−*/*−^ MEFs were 17–27% longer than WT cilia resulting in the average length of 1.54 µm in *Tsc1*^−*/*−^ cells and 1.40 µm in *Tsc2*^−*/*−^ cells, respectively [43]; in both cases, shorter than the average cilia length of 2.7 µm measured in our WT cells. The reason for this discrepancy is unknown. Hartman et al. cultured the cells for 48 h completely without serum [43], but in our system, we were not able to obtain viable cells under these conditions. We confirmed our results by demonstrating similar cilia phenotypes in cells subjected to siRNA-mediated knock down of *Tsc1* and *Tsc2*, respectively. In addition, whereas Hartman et al. [43] found that the length of the cilia was insensitive to rapamycin treatment, we found that inhibition of mTORC1 activity abrogated the long cilia phenotype in *Tsc1*^−*/*−^ MEFs. That loss of TSC1 causes elongated cilia in a rapamycin-sensitive manner has previously been reported in *Tsc1* conditional knockout distal convoluted tubules in mice [44], and in *Danio rerio* (zebrafish) [33, 51].

Increased mTORC1 activity was observed in both the *Tsc2*^−*/*−^ and *Tsc1*^−*/*−^ cells, as expected, due to lack of inhibition of mTORCI. It is also generally accepted that increased mTORC1 activity is associated with reduced autophagy [47, 48], but surprisingly we observed that loss of *Tsc1* led to increased autophagic flux, and both increased mTORC1 activity and increased autophagy was inhibited by rapamycin treatment. The increased autophagic flux in the *Tsc1*^−*/*−^ cells correlated with an increased level of LC3B, which may indicate increased autophagy as it has been shown that starvation leads to fast conversion of LC3B-I to LC3B-II (after 15–30 min) and to increased expression of LC3B after long time starvation [70]. In line with a recent study in mouse bone marrow macrophages [71], we also found that loss of *Tsc1* in MEFs leads to increased amount of LC3B protein, which can be normalized by rapamycin. In addition, *Tsc1*^−*/*−^ macrophages dispalyed  increased number of autophagosomes, activation of AMPK, phosphorylation of ULK1 (site Ser555), and increased amount of Beclin1 protein, all observations supporting increased autophagic activity in the *Tsc1*^−*/*−^ cells [71].

Several studies have shown that autophagy regulates cilia length [34, 35, 51–55], but with disagreement about whether increased autophagy leads to longer or shorter cilia. Three different studies [52, 53, 55] demonstrated that cigarette smoke, flavonoid silibinin and the anti-malaria medicine mefloquine lead to increased autophagy. However, whereas cigarette smoke and silibinin treatments was associated with  shortening of cilia in mouse lung tissue and embryonic preadipocytes [52, 55], cilia were elongated as an effect of mefloquine treatment in RPE cells [53]. Furthermore, the effect of *Atg5*, a gene essential for autophagy, was investigated in three other studies, with different outcomes [34, 35, 54]. Whereas longer cilia were observed in mutant MEFs in one study [35], shorter and reduced number of cilia were observed in two other studies in *Atg5* mutant MEFs and mice kidney proximal tubular cells, respectively [34, 54], indicating that autophagy induces cilia growth, in agreement with our results.

Further, our data show that treatment with lysosomal protease inhibitors reduces the ciliary length in both WT and mutant MEFs, and treatment with the autophagy inducer ABT-737 leads to cilia elongation in *Tsc1*^−*/*−^ cells. These observations indicate that nutrient deprivation-induced ciliogenesis depends on proteins delivered by autophagy. Furthermore, the rapamycin-sensitive long cilia phenotype observed in *Tsc1*^−*/*−^ cells indicates that active mTORC1 in these cells is a prerequisite for increased cilium elongation. This result is in agreement with previous observations of cilia in Kupffer’s vesicle (ciliated epithelial organ) of *Tsc1*^−*/*−^ zebrafish, where rapamycin treatment was shown to cause cilia shortening and overexpression of S6K1 led to ciliary lengthening [51].

**Fig. 7:**
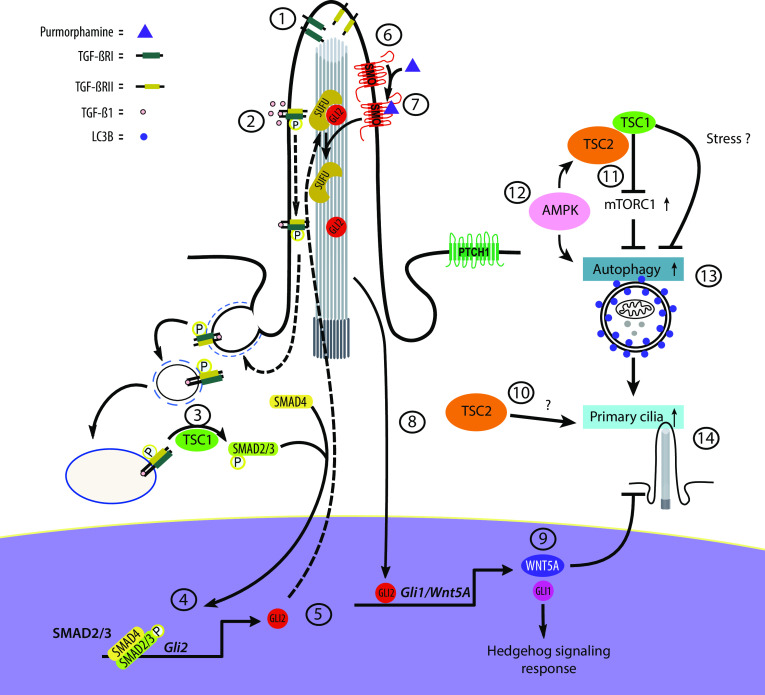
Model illustrating the effect of TSC1 on HH signaling, ciliogenesis and cilia disassembly. 1: TGF-β receptors (TGF-β-RI and TGF-β-RII) localize to the ciliary tip. 2: TGF-β1 stimulation leads to heterotetramerization of the receptors and activation by phosphorylation of SMAD2/3 at the ciliary base. 3: TSC1 is required for this step. 4: phosphorylated SMAD2/3 (pSMAD2/3) acts (in a trimeric complex with SMAD4) as transcription factor leading to expression of *Gli2*. 5: GLI2 is translocated to the cilium where it is inhibited by SUFU (negative regulator of HH). 6: inactive SMO is activated by its agonist purmorphamine. 7: activation of SMO leads to removal of SUFU followed by expression of *Gli2.* 8: active GLI2 enters the nucleus as a transcription factor leading to expression of *Gli1* and *Wnt5a*. 9: WNT5A mediates primary cilia disassembly. 10: *Tsc2*-deficient cells display shortened primary cilia. 11: the TSC1/TSC2 complex downregulates mTORC1, leading to increased autophagy. 12: during starvation AMPK promotes autophagy directly and indirectly through phosphorylation of TSC2, which then inhibit mTORC1. 13: *Tsc1*-deficient cells display increased mTORC1 activity but at the same time mTORC1-dependent increased autophagic flux. 14: autophagic activity stimulates ciliogenesis

As a result of low energy stress due to the constitutive mTORC1 activation in *Tsc1*^−*/*−^ and *Tsc2*^−*/*−^ cells, it is possible that AMPK is activated. AMPK induces autophagy, directly through phosphorylation of ULK1 (Ser555) and indirectly through phosphorylation of TSC2, which then inhibits mTORC1 [7]. The increased autophagic activity in *Tsc1*^−*/*−^ relative to *Tsc2*^−*/*−^cells might be due to the lack of TSC2 in *Tsc2*^−*/*−^ cells. The reduced autophagic activity in WT cells compared to *Tsc1*^−*/*−^ cells might be due to an intact TSC1/TSC2 complex in WT cells capable of keeping the mTOR activity under control, preventing overuse of resources. Thus, the increased level of autophagy in *Tsc1*^−*/*−^ cells compared to WT cells might be a result of constitutive mTORC1 activation, which in turn may promote ciliary extension, in agreement with the observed ciliary lengthening in the *Tsc1*^−*/*−^ cells, but not in WT cells, as a result of ABT-737-induced autophagy. Alternatively, the observed increased autophagy in *Tsc1*^−*/*−^ cells could be due to a yet unknown TSC1-dependent mechanism on autophagy (Fig. 7).

While few studies have focused on cross-talk between HH and mTORC1 signaling, the progression of epithelial to mesenchymal transition, which marks the potential invasiveness and metastatic potential of tumors, was shown to be diminished by rapamycin in a xenograft model of rhabdomyosarcoma in a manner that not only inhibited mTORC1 activity but also HH signaling [72]. Furthermore, a cross-talk between the two pathways has been described in a model of esophageal cancer where mTORC1/S6K1 promotes the oncogenic function of *GLI1,* through S6K1-mediated phosphorylation of GLI1, in a SMO-independent manner [57]. We here show that TSC1 and TSC2 via different mechanisms play critical roles in activation of canonical, SMO-dependent HH signaling adding further complexity to the role of mTOR signaling components in coordinating diverse HH pathways. In both *Tsc1*^−*/*−^ and *Tsc2*^−*/*−^ MEFs, the levels of *Gli1* and *Ptch1* expression in purmorphamine-stimulated cells were greatly reduced as compared to WT cells, but the ability to respond to purmorphamine was to a large degree restored by rapamycin only in *Tsc2*^−*/*−^ MEFs. These results suggest that the activation of canonical HH signaling relies on the inhibition of mTORC1 activity in a TSC2-dependent manner, while TSC1 largely operates through mTORC1-independent pathway(s) to ensure robust HH signaling in response to activation of SMO. Indeed, we demonstrate that impaired HH signaling in *Tsc1*^−*/*−^ MEFs, is due to low expression of *Gli2*, likely as a result of impaired TGF-β-SMAD2/3 signaling (Fig. 5a). This is in line with previous work showing that SMAD2/3 activation is impaired in *Tsc1*^−*/*−^ but not in *Tsc2*^−*/*−^ cells [11].

It is important to address the question of how and where TSC1 contributes to proper TGF-β signaling. We did not investigate expression levels and the cellular localization of TGF-β receptors, but previous studies showed that receptors and downstream signaling components in TGF-β signaling are enriched in primary cilia and at the ciliary base region [28]. In fibroblasts, TGF-β1 stimulation leads to a translocation of TGF-β receptors from the cilium to the ciliary pocket, for clathrin-mediated endocytosis of the activated TGF-β receptors, and phosphorylation of SMAD2/3 in early endosomes at the ciliary base region [28] (Fig. 7). Since TSC1 contains a putative transmembrane domain [73] and localizes to the ciliary base in human RPE cells [43] and early endosomes in HeLa cells [11], TSC1 may function at the primary cilium to regulate TGF-β receptor-mediated activation of SMAD2/3, although speculative at this point.

TGF-β-SMAD2/3 signaling has previously been associated with TSC as the expression of TGF-β ligand is upregulated in angiofibromas obtained from TSC patients [74]. However, the genotype of the TSC patients examined in this study was not specified. Furthermore, TGF-β activates mTORC1 in murine fibroblasts but not in epithelial cells via a PI3 K/AKT/TSC2-dependent pathway [75]. It remains to be investigated whether this effect on mTOR activity is compromised only in *Tsc2*^−*/*−^ MEFs.

Since TGF-β and HH signaling converge at the level of GLI2, we further elaborated on the link between low *Gli2* expression and the observed elongated ciliary length phenotype in *Tsc1*^−*/*−^ MEFs. TGF-β1-driven *Gli2* expression contributes to canonical HH signaling, but *Gli2* may also regulate target gene expression downstream of TGF-β signaling, including expression of genes encoding members of the WNT protein family [68]. In the canonical WNT pathway, binding of WNT ligands to Frizzled receptors induces β-catenin stabilization and entry into the nucleus to regulate target gene transcription. However, the so-called non-canonical WNT proteins, such as WNT5A [76], are involved in alternate signaling cascades including a role in WNT5A-PLK1-dependent stimulation of Aurora A kinase, leading to the disassembly of the primary cilium [69]. We here demonstrate that a low *Gli2* mRNA level correlated with reduced *Wnt5a* expression, which in turn contributes to an elongated ciliary length phenotype. The cilia length in the *Tsc1*^−*/*−^ cells was reduced by both recombinant WNT5A and by rapamycin treatment. Whereas reduced expression of *Wnt5a* could be explained by aberrant TGF-β-SMAD2/3 signaling, the effect of rapamycin could be explained by mTORC1-dependent increased autophagic flux in the *Tsc1*^−*/*−^ MEFs. However, we cannot exclude the possibility that rapamycin treatment also leads to even shorter cilia in *Tsc2*^−*/*−^ cells, as the cilia are already very short and difficult to measure by our microscopy setup.

In summary, we have demonstrated that loss of TSC1 causes both increased autophagic flux and impaired SMO-dependent HH signaling due to reduced expression of *Gli2*. The long cilia phenotype in *Tsc1*^−*/*−^ MEFs could be explained by a combination of increased cilia growth due to increased autophagic flux and reduced rate of disassembly due to reduced expression of the transcription factor *Gli2* that regulates the expression of *Wnt5a* (Fig. 7). Although substantial progress has been made in understanding the role of the heterodimeric complex formed by the proteins encoded by TSC1 and TSC2 in TSC pathology, many questions remain regarding the separate functions of these two genes. Addressing these questions in the future will contribute to a better understanding of the molecular etiology of TSC and potential new opportunities for therapy.

## Materials and methods

### Cell cultures and reagents

All mouse embryonic fibroblast cells were obtained from D. Kwiatkowski, Harvard University, Boston, MA, USA. They were cultured in Dulbecco’s Modified Eagle Medium (DMEM) supplemented with Glutamax, 10% Fetal Bovine Serum (FBS), 1% penicillin–streptomycin. The cells were grown in a 5% CO_2_ incubator at 37 °C. To induce ciliary formation cells were starved (0.5% FBS) for 48 h, and to measure autophagic flux cells were cultured in the presence and in the absence of rapamycin before lysosomal degradation was inhibited by 20 mM ammonium chloride (NH_4_Cl, Sigma-Aldrich #A-0171) and 200 µm Leupeptin (Biovision #1648) for 2 or 4 h. Cells were treated with 25 nM rapamycin (Cell Signaling Technology #9904) for 48 h to inhibit mTORC1 activity and 5 µM purmorphamine (Santa Cruz biotechnologies #sc-202785) for 24 h to activate HH signaling (last 24 h of the 48 h in starvation medium). To induce autophagy, cells were further incubated with 10 µM ABT-737 (Selleckchem # S1002) for 24 h. SMAD2/3 signaling was stimulated with 2 ng ml^−1^ recombinant human TGF-β1 (R&D Biosystems #240-B) and inhibited with 10 μM SB431542 (InvivoGen #inh-sb43) for indicated time points. Recombinant human/mouse WNT5a (fragment Gln38Lys380, which is identical in mouse and human expressed in Chinese Hamster Ovary cell line, R&D Biosystems #645-WN) was administered to the cells at 300 nM for 24 h prior to induction of ciliary formation in starvation medium (0.5% FBS). Primary antibodies used for IFM were as follows (dilutions, vendor and catalog number in parenthesis): mouse anti-acetylated α tubulin (1:2000, Sigma-Aldrich #T6793), rabbit anti-α-tubulin (1:1000, Sigma-Aldrich #T5192), rabbit anti-KI67 (1:250, Abcam #ab15580), rabbit anti-IFT88 (1:1000, Nordic Biosite/Proteintech, 13967-1-ap). Secondary antibodies used were Alexa fluor 546 donkey anti-mouse IgG (1:1000, Invitrogen # A10036) and Alexa fluor 488 goat anti-rabbit IgG(1:1000, Invitrogen # A11008). Dnase I (Invitrogen, 18068015) was used for degradation of plasmids present in RNA samples. The following primary antibodies were used for WB analysis (dilutions, vendor and catalog number in parenthesis): rabbit anti-phospho-S6 Ribosomal Protein (1:1000, Cell Signaling # 4858), rabbit anti-GAPDH (1:5000, Cell Signaling #2118), rabbit anti-total-S6 Ribosomal Protein (1:1000, Cell Signaling #2217), rabbit anti-TSC1 (1:500, Abcam #ab25882), rabbit anti-TSC2 (1:1000, Cell Signaling #4308), rabbit anti-phospho-SMAD2 (1:1000 Cell Signaling #3101), rabbit anti-total-SMAD2 (1:1000 Cell Signaling #5339), goat anti GLI2 antibody (1:1000 R&D system AF3635-SP), goat anti-GLI3 (1:1000 R&D system AF3690-SP) rabbit anti Lamin A antibody (1:1000, abcam ab26300), mouse anti-α-tubulin (1:5000, Sigma-Aldrich #T6199) and rabbit anti LC3B (1:250, Sigma-Aldrich #L7543. Secondary antibodies used were HRP-conjugated goat anti-mouse IgG (1:2000, DAKO, # P0447), HRP-conjugated Swine anti-rabbit IgG (1:2000, DAKO, # P0399) and HRP-conjugated rabbit anti-goat IgG (1:2000, DAKO, #Pol60).

### Immunofluorescence microscopy (IFM) and imaging analysis

Approximately 0.5 × 10^6^ cells per well were seeded in a 6-well plate containing glass coverslips and allowed to incubate in complete medium (DMEM containing 10% FBS, 1% penicillin–streptomycin) for 24 h prior to cilia induction by culturing cells in starvation medium (0.5% FBS) for 48 h followed by three times washing in ice-cold phosphate-buffered saline (PBS). They were then fixed in 4% paraformaldehyde for 15 min, washed three times in PBS, permeabilized with 1% Triton-X-100 in PBS for 15 min, and incubated in blocking solution [PBS containing 3% bovine serum albumin (BSA)] for 30 min. The cells were incubated with primary antibodies overnight at 4 °C and washed three times in blocking solution. Secondary antibodies were added for 45 min at room temperature followed by washing for 5 min in blocking solution. They were then incubated with 0.5 μg ml^−1^ 4′, 6-diamidino-2-phenylindole for 30 s to stain DNA, washed three times in PBS and mounted with an anti-fading mounting gel containing N-propyl gallate (Sigma-Aldrich #P3130). All procedures were performed at room temperature. Confocal microscopy was performed using an Olympus Fluoview 1000 FV. Adjustments to brightness and contrast were minimal and applied to the whole image. Z-stacked images were Z-projected and Fiji software (http://fiji.sc) was used for measuring cilia length.

### SDS-PAGE and WB analyses

The cells were lysed with ice-cold lysis buffer (50 mM Tris–HCl pH 6.8, 10% glycerol, 2.5% SDS, 10 mM DTE, 10 mM β-glycerophosphate, 10 mM NaF, 0.1 mM Na-orthovanadate, 1 mM phenylmethylsulfonyl fluoride and 1× complete protease inhibitor cocktail tablets (Roche #11697498001), vortexed for 10 s and left on ice for 30 min followed by 3 × 10 s. sonication, and centrifuged for 5 min at 13,000 rpm at 4 °C. Supernatant fractions were used for protein analysis. Total protein (20 μg) was separated by SDS-PAGE and transferred to nitrocellulose membranes. After applying blocking solution (100 mM Tris pH 7.6, 0.1% Tween 20, 5% dry milk) for 45 min at room temperature, the membranes were incubated with primary antibodies and left over night at 4 °C. They were then washed five times in TBS-T (100 mM Tris pH 7.6, 0.1% Tween 20), followed by incubation with HRP-conjugated secondary antibodies, which were detected with SuperSignal™ West Dura (ThermoFisher Scientific # 34076) and digitally developed by chemiluminescence using G:Box Chemi XX6 (Syngene). Where necessary, the membranes were stripped by two rinses in stripping solution (1% Tween 20, 0.1% SDS, 1.5% glycine, pH 2.2) followed by two rinses in PBS and TBS-T. The membranes were then ready to be blocked again. The optical density of the bands was analyzed with the gel analyzer of the Fiji software.

### Plasmid transfection and siRNA-mediated gene knock down

The following plasmids were used for transient plasmid transfection: GFP-tagged Gli2 (pGli2, mouse) (Addgene #37672), Myc-tagged human WT-*TSC1*, pTSC1 and human TSC2-Flag, pTSC2 (both a gift from Mark Nellist, Erasmus MC, Rotterdam). JetPRIME (Polyplus transfection, Illkirch, France) was used as the transfection reagent following the protocol provided by the manufacturer. RNA silencing was achieved using siRNA targeting mouse *Tsc1* (#1: TTGGTTGATTATTACCTGGAA #2: ATCGAGAAAGATAAGGAAGAA), *Tsc2* (#1: CAGCATTAATATCTTATCACA #2: CTGGACATCATTGAACGACTA), Wnt5a (AAGATCTTAAATATAGATATA) and *Gli2* (CACCAACCCTTCAGACTATTA) or a scramble sequence for control at a final concentration of 25 nM. All siRNAs were from Qiagen and delivered to cells using DharmaFECT1 transfection reagent (Dharmacon), following the protocol provided by the manufacturers.

### Quantitative RT-PCR analysis (qPCR)

For cDNA preparation total RNA was extracted from the cell cultures using the RNeasy Mini kit (Qiagen) following the instructions provided by the manufacturer. To validate the transfection efficiency with plasmids (p.TSC1, p.TSC2, p.Gli2), an isolated RNA was treated with DNase I (11284932001 Roche**)** before cDNA preparation according to the manufacturer’s protocol. An empty p.cDNA3 vector was used for Mock transfection. The RNA concentration was determined using a NanoDrop Spectrophotometer (Thermo Fisher Scientific). For cDNA preparation, reverse transcription was carried out using the High-Capacity cDNA Reverse Transcription Kit (Thermo Fisher Scientific). Standard qPCR was carried using predesigned TaqMan probes (Thermo Fisher Scientific) targeting the gene of interest. The measurements are performed in triplicates. The expression levels were normalized to the level of TATA binding protein (TBP) in the same samples after amplification with the TaqMan Gene Expression Master Mix (Thermo Fisher Scientific) on a 7500 Real-Time PCR system (Applied Biosystems) and the − ΔΔ*C*_t_ method was used to evaluate differential expression [77]. The *C*_t_ value is the cycle number at which the fluorescence generated within a reaction crosses the threshold line. *C*_t_ levels are inversely proportional to the amount of target in the sample, the lower the *C*_t_ level the greater the amount of target. Δ*C*_t_ is the difference in *C*_t_ values for our target gene and our housekeeping gene (*Tbp*) for a given sample (normalized values) and ΔΔ*C*_t_ is the difference in Δ*C*_t_ values between treated and untreated samples [77]. The negative value − ΔΔ*C*_t_, is used as the exponent of 2 in the equation $2^{{ - \Delta \Delta C_{\text{t}} }}$ and represents the difference in normalized number of *C*_t_ values. The exponent 2 is used based on the assumption that each cycle doubles the amount of product. If the control sample Δ*C*_t_ is 4 and the treated sample Δ*C*_t_ is 2, then $2^{{ - \Delta \Delta C_{\text{t}} }}$ (2^−(2–4)^) yields 4, meaning that the gene of interest in the treated sample is increased four times in comparison to the control sample.

### Cellular fractionation

Cellular fractionation was performed as described previously [78].

### Statistical analysis

Quantitative results represent the mean of at least three independent experiments, if not specified otherwise. Error bars represent standard error of the mean (SEM, *n* ≥ 3). *p* values were calculated using Student’s *t* test, if not specified otherwise. *p < 0.05, **p < 0.01, ***p < 0.001, ****p < 0.0001). ¤: borderline value *p* = 0.055.

### Electronic supplementary material

Below is the link to the electronic supplementary material.

Supplementary material 1 (PDF 582 kb)

## Abbreviations

HH: Hedgehog
WNT: Wingless/Int
TGF-β: Transforming growth factor-β
TSC: Tuberous sclerosis complex
MEFs: Mouse embryonic fibroblasts
4E-BP1: 4E-binding protein-1
S6K1: 40S ribosomal protein S6 kinase 1
PTCH1: Patched homolog 1
SMO: Smoothened
