# Supplementary material for: TSC1 and TSC2 regulate cilia length and canonical Hedgehog signaling via different mechanisms
Source: Cell Mol Life Sci. 2018 Feb 2;75(14):2663–80. doi: 10.1007/s00018-018-2761-8 (PMC6003990; doi:10.1007/s00018-018-2761-8)
Supplement: Supplementary file 1 — Supplementary material 1 (PDF 582 kb) [file 18_2018_2761_MOESM1_ESM.pdf]

Supplementary table and figures to CMLS-D-17-00236:

TSC1 and TSC2 regulate cilia length and canonical Hedgehog signaling via different mechanisms

Thomas Rosengren[1,γ], Lasse Jonsgaard Larsen[1,γ], Lotte Bang Pedersen[2], Søren Tvorup Christensen[2] and Lisbeth Birk Møller[1,*]

***Corresponding author**: Lisbeth.Birk.Moeller@regionh.dk, telephone +45 43260130
[γ]These authors contributed equally to this work.

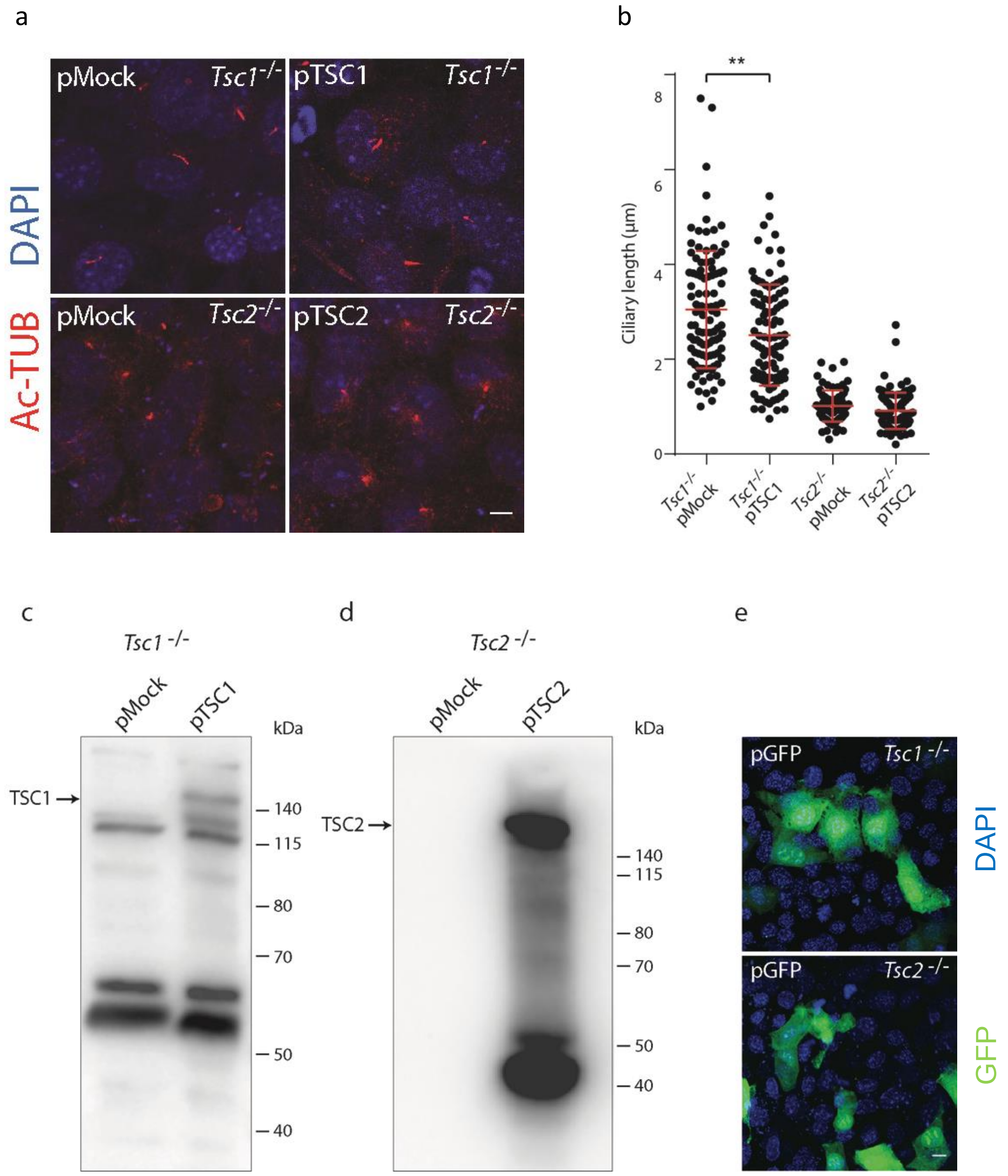

Supplementary Figure 1

Exogenous expression of *TSC1* and *TSC2* in MEFs. (a) Investigation by IFM of cilia in *Tsc1*$^{-/-}$ and *Tsc2*$^{-/-}$ MEFs after transfection with pTSC1 expressing the human *TSC1* gene or pTSC2 expressing the human *TSC2* gene, respectively, or transfection with pMock (empty pcDNA3 vector). Twenty four hrs after transfection, the MEFs were cultured in low serum medium (0.5 % FBS) for 48 hrs to induce cilia formation before fixation and incubation with anti-acetylated α-tubulin (Ac-TUB) antibody. The nuclei were stained with DAPI. Scalebar: 5 µM. (b) Quantification of cilia length for experiment shown in (a). 100 cilia were measured per condition in this quantification. (c) SDS-PAGE and WB of cell lysate from *Tsc1*$^{-/-}$ MEFs transfected with pTSC1, or pMock. The blot was incubated with antibody against TSC1. (d) Cell lysate from *Tsc2*$^{-/-}$ MEFs transfected with pTSC2 or pMock. The blot was incubated with antibody against TSC2. (e) Investigation by IFM of *Tsc1*$^{-/-}$ and *Tsc2*$^{-/-}$ MEFs after transfection with pGFP plasmid. The transfection efficiency was at least 15% in *Tsc1*$^{-/-}$ and 14% *Tsc2*$^{-/-}$ MEFs, respectively. More than 500 cells of each type were analyzed for this quantification. Scalebar: 10 µM.

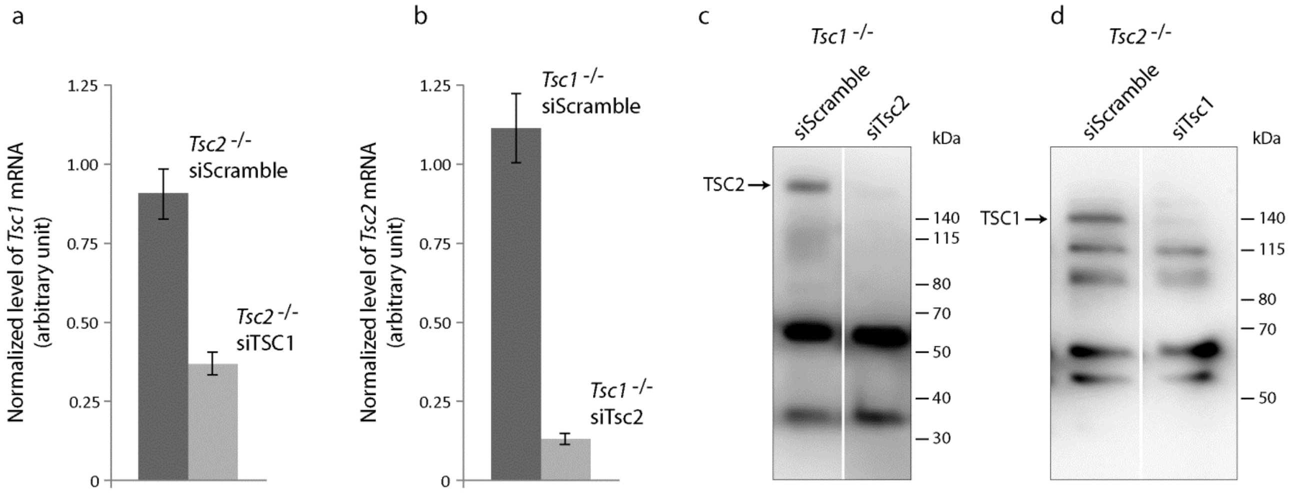

Supplementary Figure 2

siRNA-mediated *Tsc1/2* gene knock down in MEFs. (a) Expression level of *Tsc1* mRNA, normalized to *Tbp* mRNA, in *Tsc2*$^{-/-}$ MEFs transfected with siTsc1 (knockdown of *Tsc1*) or siScramble (negative control). (b) Expression level of *Tsc2* mRNA, normalized to *Tbp* mRNA, in *Tsc1*$^{-/-}$ MEFs transfected with siTsc2 (knockdown of *Tsc2*) or siScramble. (c) WB analysis of TSC2 protein in cell lysate from *Tsc1*$^{-/-}$ MEFs transfected with siTsc2 or siScramble using TSC2 antibody. (d) WB analysis of TSC1 protein in cell lysate from *Tsc2*$^{-/-}$ cells transfected with siTsc1 or siScramble using TSC1 antibody.

a

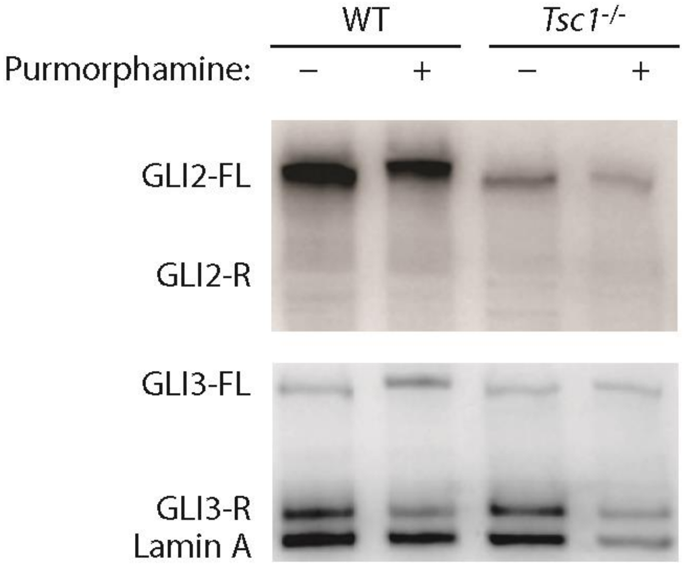

b

Cell type	Gli3-FL/Gli3-R - Pur	Gli3-F/Gli3-R +Pur
WT	0.481 ± 0.226	0.828 ± 0.220
Tsc1$^{-/-}$	0.435 ± 0.222	0.590 ± 0.487

Supplementary Figure 3

Western blot analysis of GLI2 and GLI3 proteins in nuclear fractions. (a) WT and *Tsc1*$^{-/-}$ cells were grown in low serum medium (0.5% FBS) for 48 hrs and incubated with or without Purmorphamine (5 μM) for the last 24 hrs before harvesting. Isolated nuclear fractions were analysed by WB. The blot was incubated with antibodies against Gli2, Gli3 and Lamin A (loading control) as indicated. The locations of GLI2-FL, GLI2-R, GLI3-FL and GLI3-R are indicated. (b) The ratios of GLI3-FL/GLI3-R were calculated from 5-6 samples per condition from 2 independent experiments. In the WT cells a significant increase in the GLI3-FL/GLI3-R ratio (p=0.039) was detected after Purmorphamine treatment (+Pur). In contrast, no significant increase in the GLI3-FL/GLI3-R ratio (p=0.50) was detected in Purmorphamine-treated *Tsc1*$^{-/-}$ cells.

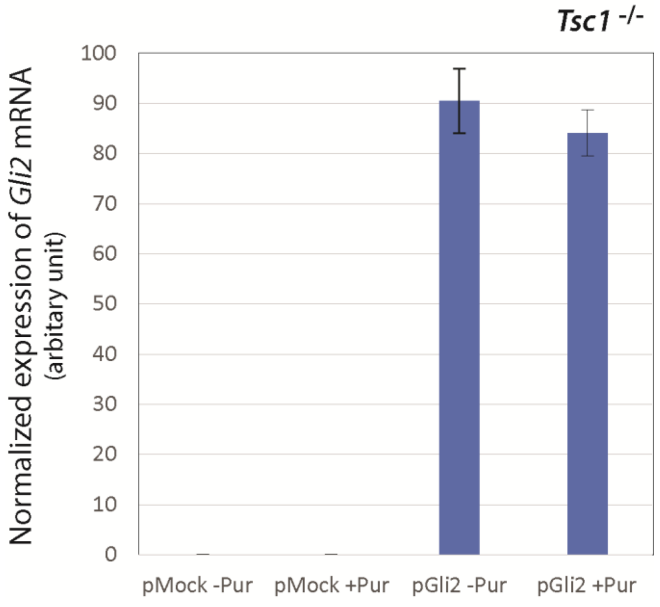

Supplementary Figure 4

Investigation of exogenous *Gli2* gene expression by qPCR. Expression of *Gli2* mRNA, normalized to *Tbp* mRNA, in *Tsc1*$^{-/-}$ MEFs transfected with pGli2, expressing the murine *Gli2* gene, or pMock (negative control, empty pcDNA3 vector). After transfection the cells were starved (0.5% FBS) for 48 hrs and incubated with or without Purmorphamine (5 µM, Pur) for the last 24 hrs. Isolated RNA was DNase I treated before cDNA preparation and qPCR. Error bars represent SEM.

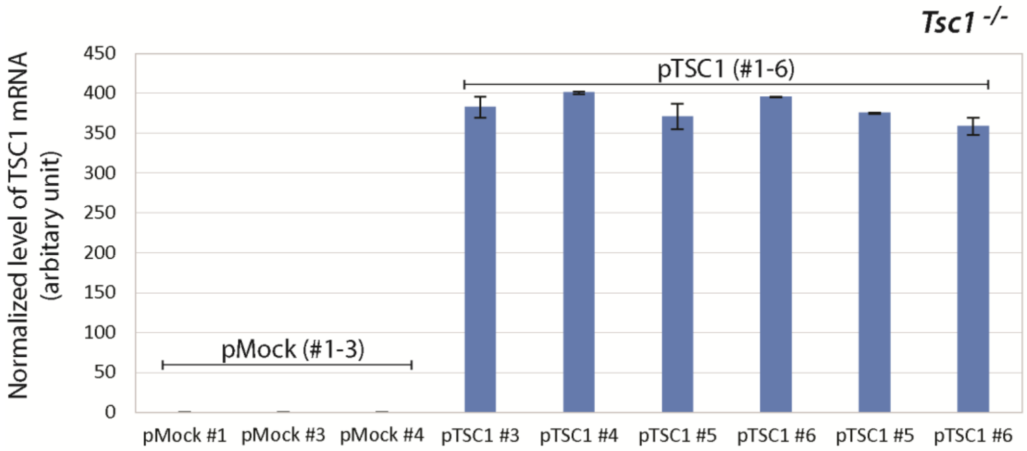

Supplementary Figure 5

Investigation of exogenous *TSC1* gene expression by qPCR. Expression of *TSC1* mRNA, normalized to *Tbp* mRNA, in *Tsc1*$^{-/-}$ MEFs transfected with pTSC1, expressing the human *TSC1* gene (6 different transfections), or pMock (empty vector) (3 different transfections, negative control). Isolated RNA was DNase I treated before cDNA preparation and qPCR. Error bars represent SEM.

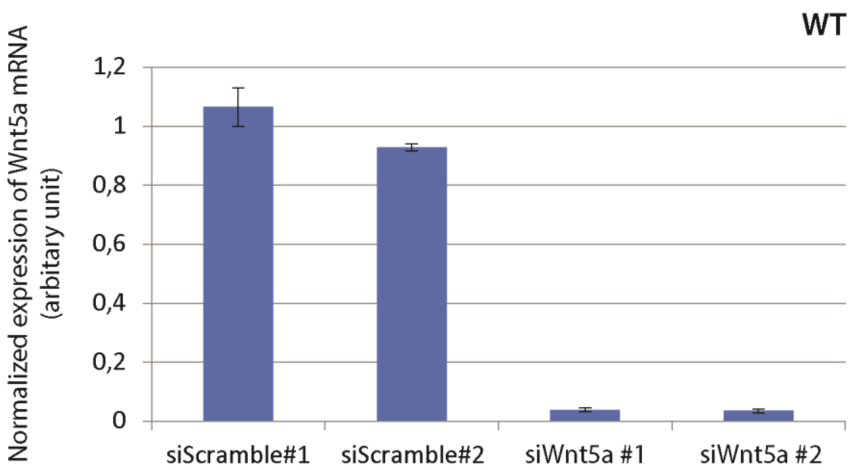

Supplementary Figure 6

Investigation of siRNA-mediated *Wnt5a* gene knock down by qPCR. Expression level of *Wnt5a* mRNA, normalized to *Tbp* mRNA, in WT cells transfected with siWnt5a or siScramble (negative control). The figure shows results from two independent experiments with different constructs. Error bars represent SEM.

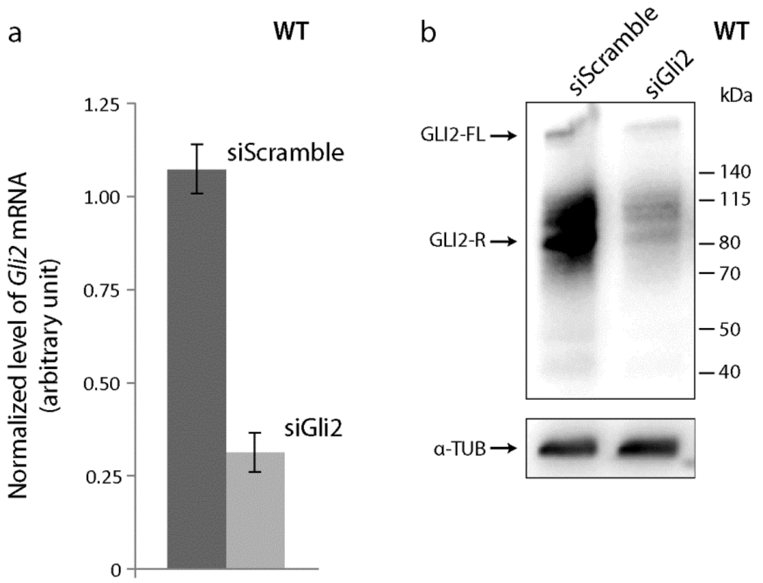

Supplementary Figure 7

siRNA-mediated *Gli2* gene knock down. (a) Expression level of *Gli2* mRNA, normalized to *Tbp* mRNA, in WT cells transfected with siGli2 or siScramble (negative control). (b) Investigation of GLI2 protein in cell lysate from WT cells transfected with siGli2 or siScramble (negative control). Antibodies against GLI2 and Tubulin (α-TUB) were used as indicated. The GLI2 antibody recognizes both the full length activator (GLI2-FL) and truncated repressor (GLI2-R) form of GLI2.
